# A comparative study of the “superior mesenteric artery first” approach versus the conventional approach in short-term and long-term outcomes in patients with pancreatic ductal adenocarcinoma undergoing laparoscopic pancreaticoduodenectomy

**DOI:** 10.1007/s00464-023-10470-7

**Published:** 2023-10-27

**Authors:** Xiaoxiang Wang, Qilan Luo, Shizhen Li, Yi Wu, Tingting Zhen, Feng Zhu, Min Wang, Shutao Pan, Renyi Qin

**Affiliations:** Department of Biliary-Pancreatic Surgery, Affiliated Tongji Hospital, Tongji Medical College, Huazhong University of Science and Technology, No. 1095 Jiefang Avenue, Wuhan, 430030 Hubei China

**Keywords:** Laparoscopic pancreaticoduodenectomy, Superior mesenteric artery, Pancreatic ductal adenocarcinoma, Long-term survival

## Abstract

**Background:**

The use of laparoscopic pancreaticoduodenectomy (LPD) in pancreatic head cancer remains controversial, and an appropriate surgical approach can help improve perioperative safety and oncological outcomes. This study aimed to assess the short-term outcomes and long-term survival of the superior mesenteric artery first (SMA-first) approach in patients with pancreatic ductal adenocarcinoma (PDAC) undergoing LPD.

**Methods:**

The data of 91 consecutive PDAC patients who underwent LPD from June 2014 to June 2021 were retrospectively analyzed. Patients were divided into two groups, the modified SMA-first approach group, using a combined posterior and anterior approach, and the conventional approach group. Perioperative outcomes, pathologic results, and overall survival (OS) were compared between groups, and propensity score-matched (PSM) analysis was performed.

**Results:**

The number of lymph nodes harvested was greater in the SMA-first approach group (19 vs. 15, *P* = 0.021), as did the results in the matched cohort (21 vs. 15, *P* = 0.046). No significant difference was observed in the R0 resection rate (93.3% vs. 82.6%, *P* = 0.197), but the involvement of the SMA margin was indeed lower in the SMA-first approach group (0 vs. 13%). There were no obvious variances between the two groups in terms of intraoperative bleeding, operative time, overall and major postoperative complication rates, and mortality in either the original cohort or matched cohort. The median OS was 21.8 months in the SMA-first group, whereas it was 19.8 months in the conventional group (*P* = 0.900). Survival also did not differ in the matched cohort (*P* = 0.558). TNM stage, resection margin, overall complications, and adjuvant therapy were independent risk factors affecting OS.

**Conclusion:**

The modified SMA-first approach is safe and feasible for PDAC patients undergoing LPD. It had a slight advantage in specimen quality, but OS was not significantly prolonged.

**Graphical abstract:**

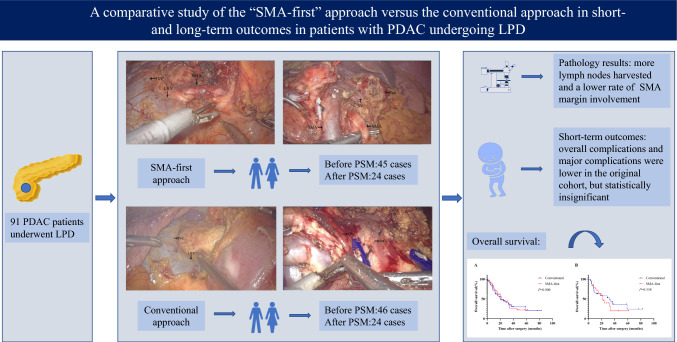

**Supplementary Information:**

The online version of this article (10.1007/s00464-023-10470-7) contains supplementary material, which is available to authorized users.

For nearly two decades, with the updates in medical equipment and the advancement of surgical technology, laparoscopic pancreaticoduodenectomy (LPD) is gradually favored by pancreatic surgeons [1]. Studies have reported that LPD is safe and reliable compared with open pancreaticoduodenectomy (OPD) and has certain advantages in reducing intraoperative bleeding, shortening hospital stay and postoperative fasting time, and reducing postoperative pain [2–4]. However, the use of LPD in pancreatic head cancer continues to receive widespread attention, mainly focusing on oncologic benefits and perioperative safety [5].

Due to the special anatomical location of the pancreatic uncinate process and the abundant surrounding blood supply, how to properly handle these vessels to reduce intraoperative bleeding remains one of the technological difficulties of LPD [6]. For pancreatic head cancer, the complete total mesopancreas excision (TMpE) to achieve adequate eradication of the tumor is the focus of attention, and it is particularly pivotal to select an appropriate surgical approach during the uncinate process of resection [7]. The superior mesenteric artery (SMA) first approach has now been used for OPD, and a meta-analysis reported that the SMA-first approach reduced blood loss, decreased overall complication rate, improved R0 resection rate, and prolonged survival in pancreatic cancer patients compared to the conventional superior mesenteric vein (SMV) approach [8]. Nevertheless, few studies have reported the results of the SMA-first approach in LPD for pancreatic head cancer [9–12]. Nagakawa et al. summarized four artery-first approaches for LPD based on the orientation during dissection of the SMA, namely the anterior approach, posterior approach, right approach, and left approach [13]. To our knowledge, to date, no article has reported the long-term survival of the SMA-first approach in LPD for pancreatic head cancer.

Thus, this study was designed to introduce a modified SMA-first approach at our center and reported its short- and long-term outcomes compared to the conventional approach in patients with pancreatic ductal adenocarcinoma (PDAC) who underwent LPD.

## Materials and methods

### Patients

**Fig. 1:**
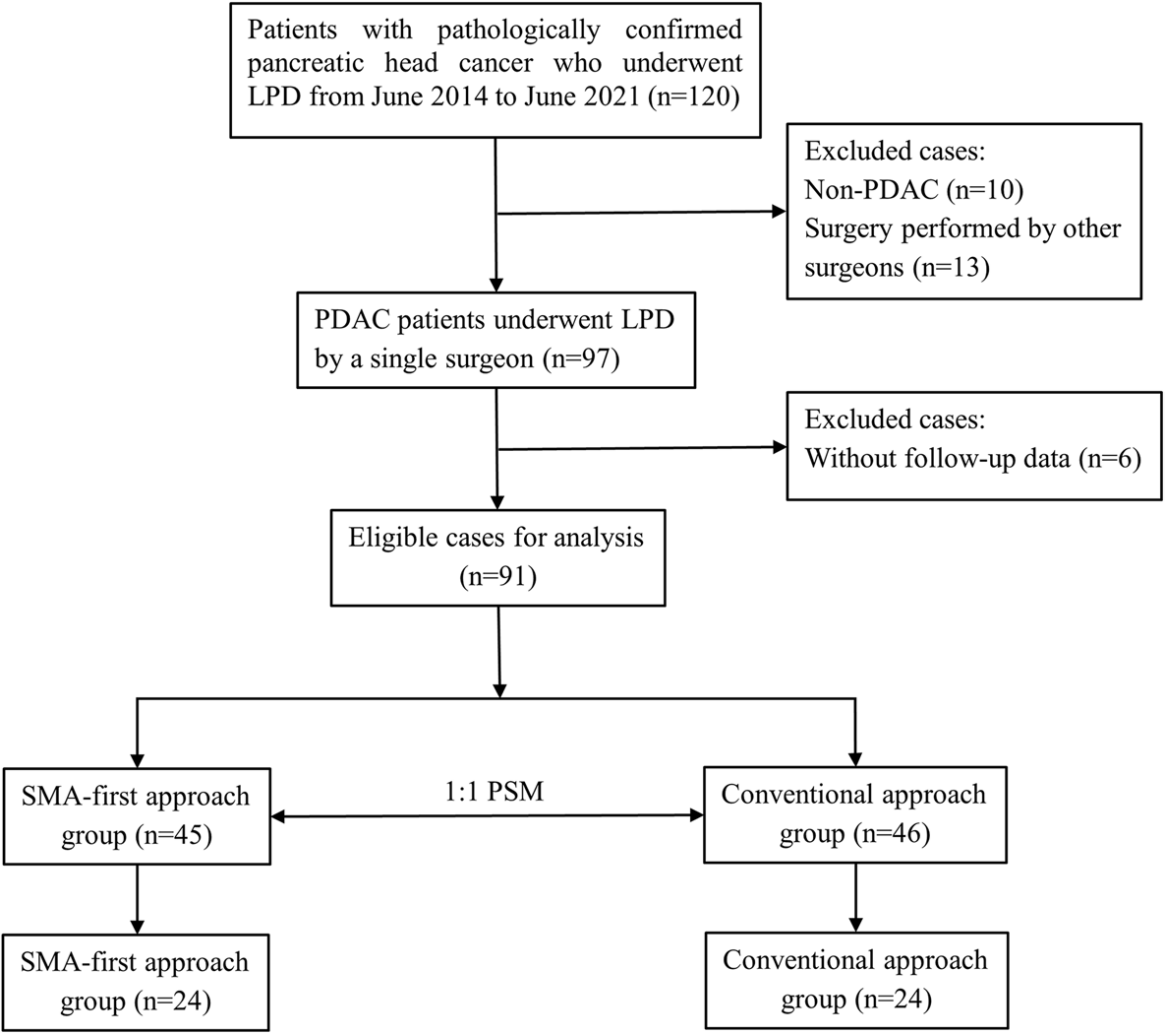
Flow chart of case screening

This retrospective cohort study collected data from 91 consecutive patients who underwent LPD at our center from June 2014 to June 2021, and all of them were pathologically diagnosed as PDAC. These 91 patients with PDAC did not receive neoadjuvant therapy. The exclusion criteria were non-PDAC and incomplete follow-up data. All LPDs were conducted by an individual surgeon who is proficient in laparoscopic pancreatic surgery. The patients were classified into two groups based on surgical modality: the SMA-first approach group (45 cases) and the conventional approach group (46 cases). Given that it was a retrospective study, the Ethics Committee of Tongji Hospital waived approval, and written consent was signed by all patients. The flow chart is visible in Fig. 1.

### Data collection and definitions

The perioperative information was retrospectively collected and analyzed. Preoperative information consisted of gender, age, American Society of Anesthesiologists (ASA) classification, body mass index (BMI), previous history (abdominal surgery, smoking, diabetes mellitus, and hypertension), preoperative biliary drainage or not, and size of tumor diameter measured by enhanced thin-laminar computed tomography. Laboratory blood results consisted of total bilirubin, carbohydrate antigen 19-9 (CA19-9), and carcinoembryonic antigen (CEA) values at admission. Intraoperative information consisted of the year of surgery, conversion to laparotomy, intraoperative bleeding, operative time, intraoperative blood transfusion, and combined vascular resection. Postoperative information consisted of the length of hospital stay, postoperative hospital stay, 30-day mortality rate, postoperative morbidities [postpancreatectomy hemorrhage (PPH), delayed gastric emptying (DGE), biliary fistula, postoperative pancreatic fistula (POPF), abdominal infection, cardiopulmonary related complications], and whether a second operation was performed. The evaluation of PPH, POPF, and DGE was based on the guidelines developed by the International Study Group of Pancreatic Surgery [14–16]. The severity of morbidities was graded by the Clavien–Dindo classification [17]. Pathological data included TNM staging according to the 8th version of the American Joint Committee on Cancer (AJCC), tumor differentiation, R0 resection rate, margin status (bile duct margin, posterior edge of the uncinate process margin, pancreatic neck margin, SMV margin, SMA margin), lymph nodes harvested, and the number of positive lymph nodes. All specimens were judged by the protocol proposed by Verbeke [18]. R0 resection was defined as a margin greater than 1 mm from the carcinoma [19].

Follow-up, including information on survival, postoperative functional disorders (diarrhea and abdominal pain), and type and application of adjuvant therapy, was conducted by telephone, outpatient, or inpatient review. Follow-ups were done one month after surgery, every 3 months for the first 2 years, and semi-annually thereafter. The most recent follow-up was on February 10, 2023. The date of surgery to the date of death or last follow-up was considered as overall survival (OS).

### Operative technique

The patient’s position was supine, and the trocar was inserted by the 5-hole way, with a 10 mm trocar put under the umbilicus for observation, a 12 mm trocar put above the umbilicus on the right midclavicular line for operation, and a 12 mm trocar above umbilicus on the left midclavicular line and 5 mm trocar below the right and left anterior rib margins as the auxiliary operation holes respectively. During the operation, the liver, great omentum, mesentery, and pelvis were first explored to exclude the presence of metastases.

#### Modified SMA-first approach

**Fig. 2:**
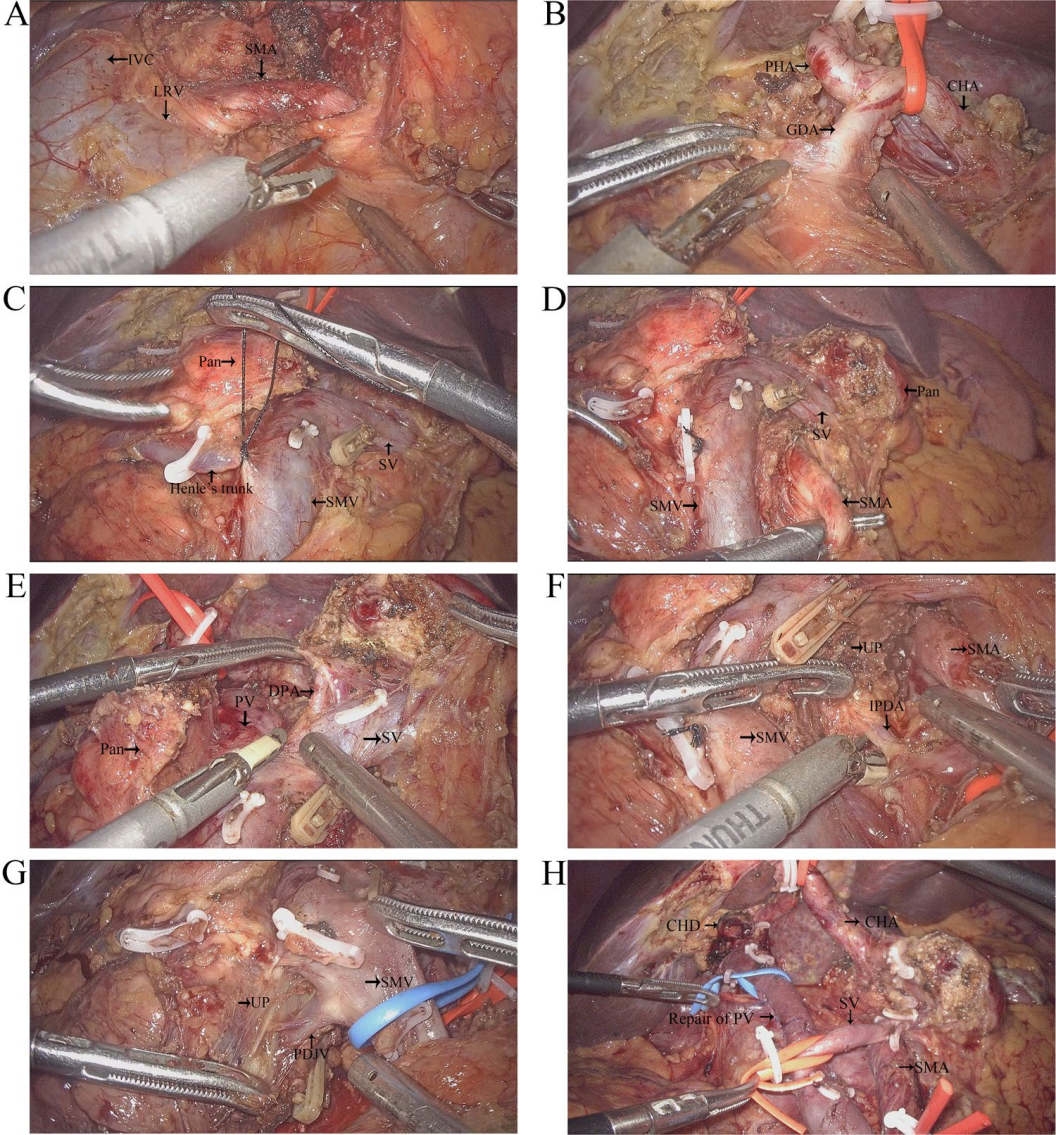
Operative technique. **A** Kocher maneuver reveals the SMA via posterior approach, **B** suspend the CHA and dissection of GDA, **C** sever the pancreatic neck, **D** the SMA is exposed on the left side of SMV via anterior approach, **E**, **F** excision of the mesopancreas and ligation of the IPDA, **G**, **H** the specimen only attached to the SMV/PV and repair of PV. *IVC* inferior vena cava, *LRV* left renal vein, *SMA* superior mesenteric artery, *CHA* common hepatic artery, *PHA* proper hepatic artery, *GDA* gastroduodenal artery, *SMV* superior mesenteric vein, *PV* portal vein, *SV* splenic vein, *DPA* dorsal pancreatic artery, *IPDA* inferior pancreaticoduodenal artery, *PDJV* proximal–dorsal jejunal vein, *CHD* common hepatic duct, *Pan* pancreas, *UP* uncinate process

After releasing the hepatic flexure of the colon, the inferior vena cava (IVC) and left renal vein (LRV) were revealed by the Kocher maneuver. The duodenum and head of the pancreas were separated from the retroperitoneum along the dimension of the fused fascia (partially requiring combined resection of Gerota fascia). Above the LRV, the peri-arterial plexus of the SMA was dissected immediately above the membrane of the artery to its root, exposing the beginning of the SMA (suspending the SMA if necessary, Fig. 2A). The gastrocolic ligament was dissected to reveal the front aspect of the pancreas head, and the horizontal part of the duodenum and the transverse colonic mesentery were separated to reveal the SMV at the inferior margin of the pancreas. The common hepatic artery (CHA) was dissected and suspended at the superior margin of the pancreas, the right gastric and gastroduodenal arteries (GDA) were ligated and severed, the CHA was skeletonized to its root over the celiac trunk (CT, Fig. 2B). Freeing the greater and lesser curvatures of the stomach and severing the stomach. The hilar part of the liver was separated and the proper hepatic artery (PHA) was skeletonized toward the end. After the transection of the common hepatic duct (CHD), the portal vein (PV) was free and suspended, and all lymph nodes within the hepatoduodenal ligament were completely cleared. The pancreatic neck was cut with an ultrasonic scalpel anteriorly or to the left of the SMV or PV, and the pancreatic duct was cut with scissors, then ligated the Henle’s trunk (Fig. 2C). The SMA was exposed and suspended to the left of the SMV at the level of the duodenum or at the mesenteric root of the transverse colon (Fig. 2D). The jejunum was severed 10 cm from the Treitz’s ligament and the proximal jejunum was pulled to the right side of the mesenteric root via the posterior aspect of the SMV and SMA. If the dorsal pancreatic artery (DPA) originated from the splenic artery (SA) was found, it was dissected together (Fig. 2E). The proximal jejunum was pulled upward to the right and the SMA was pulled upward to the left. The SMA sheath was dissected longitudinally, the uncinate mesopancreas was dissected along the anterior, right, and posterior edges of the SMA adventitia, and the inferior pancreaticoduodenal artery (IPDA) was dissected in the process (Fig. 2F). Next, the nerve and connective tissue between the SMA and the uncinate process were disconnected along the root of the SMA to the right of the CT. At this point, the arterial blood supply to the uncinate process was thoroughly disconnected, and only the SMV or PV was connected to the resected tissue. If the SMV was invaded, segmental vessel resection or vessel lateral resection could be performed, or if not invaded, the uncinate process could be gradually separated from the SMV or PV (Fig. 2G, H).

#### Conventional approach

In this approach, the SMA was not routinely explored. The uncinate process was handled around the SMV/PV axis, and the uncinate process was separated from the right side of the SMV and SMA from the caudal to the cephalic side, from anterior to posterior, from superficial to deep, layer by layer.

The sequence of digestive tract reconstruction was performed using the Child method, with an abdominal drainage tube placed next to the pancreaticojejunostomy and hepaticojejunostomy.

### Statistical analysis

An intention-to-treat and propensity score-matched (PSM) analysis based on baseline characteristics (age, sex, BMI, ASA class, abdominal surgery, smoking, diabetes, hypertension, presence of obstructive jaundice, CA19-9, CEA, year of surgery, tumor size measured on preoperative imaging) using the 1:1 nearest-neighbor method was performed in this study, and the caliper value was set to 0.1. The continuous variables were presented as mean ± standard deviation or median (P25–P75), with independent sample *t*-test or Mann–Whitney *U* test used for further intergroup comparison in the original cohort, and paired sample *t*-test and Wilcoxon signed rank sum test in the matched cohort. Categorical variables were presented as numbers (percentage), with the chi-square test or Fisher’s exact test used for further intergroup comparison in the original cohort, and the McNemar test in the matched cohort. Univariate and multivariate logistic regression analyses were used to determine the independent risk factors affecting overall complications. The Kaplan–Meier method was used to plot survival curves, and COX univariate and multivariate regression analyses were used to identify independent risk factors affecting OS. *P* < 0.05 two-sided test was statistically significant. All statistical analyses were done with SPSS 27.0 (IBM, Armonk, NY, USA) and GraphPad Prism 9.

## Results

### Baseline characteristics

Table 1 showed the baseline data of the PDAC patients. After PSM analysis, there were 24 patients in each of the SMA-first and conventional groups. The original cohort consisted of 91 patients, with 53 males and 38 females, and a mean age of 59.6 ± 8.7 years. The number of patients with obstructive jaundice was higher in the conventional approach group (87.0% vs. 55.6%, *P* = 0.001), but no significant difference was observed between intergroup in the proportion of preoperative biliary drainage (65.2% vs. 48.9%, *P* = 0.141). No significant difference in other baseline data was found, and all baseline data were balanced and comparable between the two groups after PSM.

**Table 1:** Baseline characteristics of PDAC patients undergoing LPD before PSM and after PSM

Variables	Before PSM			After PSM		
	SMA-first approach (*n* = 45)	Conventional approach (*n* = 46)	*P* value	SMA-first approach (*n* = 24)	Conventional approach (*n* = 24)	*P* value
Age (years)			0.402			1.000
> 60	27 (60.0)	23 (50.0)		12 (50.0)	11 (45.8)	
≤ 60	18 (40.0)	23 (50.0)		12 (50.0)	13 (54.2)	
Sex			1.000			0.774
Male	26 (57.8)	27 (58.7)		15 (62.5)	13 (54.2)	
Female	19 (42.2)	19 (41.3)		9 (37.5)	11 (45.8)	
BMI (kg/m^2^)			0.303			1.000
≥ 24	7 (15.6)	12 (26.1)		4 (16.7)	3 (12.5)	
< 24	38 (84.4)	34 (73.9)		20 (83.3)	21 (87.5)	
ASA class			1.000			1.000
I/II	37 (82.2)	37 (80.4)		20 (83.3)	20 (83.3)	
III	8 (17.8)	9 (19.6)		4 (16.7)	4 (16.7)	
Previous history						
Abdominal surgery	12 (26.7)	7 (15.2)	0.206	5 (20.8)	4 (16.7)	1.000
Smoking	9 (20.0)	8 (17.4)	0.793	4 (16.7)	4 (16.7)	1.000
Diabetes	8 (17.8)	2 (4.3)	0.050*	2 (8.3)	2 (8.3)	1.000
Hypertension	9 (20.0)	11 (23.9)	0.801	6 (25.0)	7 (29.2)	1.000
Obstructive jaundice	25 (55.6)	40 (87.0)	0.001	17 (70.8)	19 (79.2)	0.625
Preoperative biliary drainage	22 (48.9)	30 (65.2)	0.141	14 (58.3)	16 (66.7)	0.754
CA19-9 (U/mL)			0.321			0.727
> 37	37 (82.2)	33 (71.7)		18 (75.0)	20 (83.3)	
≤ 37	8 (17.8)	13 (28.3)		6 (25.0)	4 (16.7)	
CEA (ng/mL)			0.431			1.000
> 5	10 (22.2)	7 (15.2)		3 (12.5)	4 (16.7)	
≤ 5	35 (77.8)	39 (84.8)		21 (87.5)	20 (83.3)	
Year of surgery			1.000			0.581
2014–2017	24 (53.3)	24 (52.2)		13 (54.2)	16 (66.7)	
2018–2021	21 (46.7)	22 (47.8)		11 (45.8)	8 (33.3)	
Tumor size (cm)	2.6 (2.0–3.5)	2.5 (2.0–3.0)	0.488	2.8 (2.0–3.4)	2.5 (2.0–3.0)	0.723

Data were expressed as *n* (%) or median (range), unless otherwise marked

*BMI* body mass index, *ASA* American Society of Anesthesiologists, *CA19-9* carbohydrate antigen 19-9, *CEA* carcinoembryonic antigen

*Fisher exact TEST

### Pathologic results

The pathological results were shown in Table 2. The differences in pathological stage and histological differentiation between the intergroup of PDAC patients were not statistically significant before and after PSM analysis. Notably, more lymph nodes were harvested in the SMA-first approach group than that in the conventional approach group (19 vs. 15, *P* = 0.021), and the results were similar in the matched cohort (21 vs. 15, *P* = 0.046). Although no significant difference in R0 resection percentage was found between the intergroup (93.3% vs. 82.6%, *P* = 0.197), the positive percentage of SMA margin was lower in the SMA-first approach group compared with the conventional approach group when considering the involved margins (0 vs. 13.0%). There was also no difference in the R0 resection percentage in the matched cohort (87.5% vs. 87.5%, *P* = 1.000), but the SMA-first approach group did have a lower proportion of positive SMA margin (0 vs. 8.3%). In the SMA-first approach group, five patients had vessel lateral wall resection and repair, and one patient underwent partial vascular resection with end-to-end anastomosis. While in the conventional group, only two patients had vessel lateral wall resection.

**Table 2:** Pathologic results of PDAC patients undergoing LPD before PSM and after PSM

Variables	Before PSM			After PSM		
	SMA-first approach (*n* = 45)	Conventional approach (*n* = 46)	*P* value	SMA-first approach (*n* = 24)	Conventional approach (*n* = 24)	*P* value
Staging (AJCC 8th)			0.897*			0.682
I	28 (62.2)	28 (60.9)		16 (66.7)	18 (75.0)	
II	13 (28.9)	15 (32.6)		6 (25.0)	5 (20.8)	
III	4 (8.9)	3 (6.5)		2 (8.3)	1 (4.2)	
T stage (AJCC 8th)			1.000*			0.924
T1	15 (33.3)	16 (34.8)		8 (33.3)	7 (29.2)	
T2	27 (60.0)	26 (56.5)		14 (58.3)	14 (58.3)	
T3	3 (6.7)	4 (8.7)		2 (8.3)	3 (12.5)	
N stage (AJCC 8th)			0.843*			0.445
N0	28 (62.2)	31 (67.4)		16 (66.7)	20 (83.3)	
N1	13 (28.9)	12 (26.1)		6 (25.0)	3 (12.5)	
N2	4 (8.9)	3 (6.5)		2 (8.3)	1 (4.2)	
Differentiation			0.522			0.607
Poor	16 (35.6)	20 (43.5)		9 (37.5)	12 (50.0)	
Moderate to well	29 (64.4)	26 (56.5)		15 (62.5)	12 (50.0)	
Number of lymph nodes	19 (15–24)	15 (13–19)	0.021	21 (15–25)	15 (13–20)	0.046
Number of positive lymph nodes	0 (0–2)	0 (0–1)	0.458	0 (0–1)	0	0.218
Resection margin			0.197			1.000
R0	42 (93.3)	38 (82.6)		21 (87.5)	21 (87.5)	
R1	3 (6.7)	8 (17.4)		3 (12.5)	3 (12.5)	
Resection margin involvement						
BDM	0	0		0	0	
PNM	1 (2.2)	0		1 (4.2)	0	
PUPM	1 (2.2)	2 (4.3)		1 (4.2)	1 (4.2)	
SMVM	2 (4.4)	3 (6.5)		2 (8.3)	1 (4.2)	
SMAM	0	6 (13.0)		0	2 (8.3)	
SMV/PV resection			0.185*			–
Vessel lateral wall resection	5 (11.1)	2 (4.3)		3 (12.5)	0	
Segmental vessel resection	1 (2.2)	0		1 (4.2)	0	

Data were expressed as *n* (%) or median (range), unless otherwise marked

*AJCC* American Joint Committee on Cancer, *BDM* bile duct margin, *PNM* pancreatic neck margin, *PUPM* posterior surface of the uncinate process margin, *SMVM* superior mesenteric vein margin, *SMAM* superior mesenteric artery margin, *SMV* superior mesenteric vein, *PV* portal vein

*Fisher exact TEST

### Short-term outcomes

No significant differences were observed in the short-term outcomes between the intergroup before and after PSM analysis, as presented in Table 3. Four patients underwent conversion to laparotomy, all of which occurred in the conventional approach group (0 vs. 8.7%, *P* = 0.117). The reasons for conversion were as follows: two patients had obvious adhesion in the abdominal cavity and severe tissue adhesion in the pancreatic head area; one patient suffered from uncontrollable bleeding during surgery; one patient had intraoperative hypercapnia, and it was considered that the patient had difficulty tolerating pneumoperitoneum. The operative time (310 vs. 300 min, *P* = 0.738) and intraoperative bleeding (100 vs. 175 ml, *P* = 0.504) were similar in the intergroup. Although the overall complications percentage (20.0% vs. 39.1%, *P* = 0.066) and major complications percentage (4.4% vs. 13.0%, *P* = 0.267) were less in the SMA-first approach group than that in the conventional approach group, statistical significance was not reached. The rates of overall complications (25.0% vs. 29.2%, *P* = 1.000) and major complications (4.2% vs. 4.2%, *P* = 1.000) in the matched cohort differed little between the two groups. Within 30 days after surgery, a total of three patients died, with one in the SMA-first approach group due to severe arrhythmias, and two in the conventional approach group. One of the two patients died due to a grade C pancreatic fistula combined with abdominal infection secondary to late postoperative hemorrhage, while the other died of grade C pancreatic fistula combined with arrhythmia after surgery. At the first postoperative follow-up, diarrhea was not more common in the SMA-first approach than in the conventional approach group (4.5% vs. 6.8%, *P* = 1.000).

**Table 3:** Short-term outcomes of PDAC patients undergoing LPD before PSM and after PSM

Variables	Before PSM			After PSM		
	SMA-first approach (*n* = 45)	Conventional approach (*n* = 46)	*P* value	SMA-first approach (*n* = 24)	Conventional approach (*n* = 24)	*P* value
Conversion	0	4 (8.7)	0.117*	0	1 (4.2)	–
Estimated blood loss (mL)	100 (50–275)	175 (73–325)	0.504	200 (50–375)	100 (85–200)	0.265
Blood transfusion (U)	0 (0–1.5)	0 (0–0.5)	0.996	0 (0–2)	0 (0–1.5)	0.720
Operative time (min)	310 (270–379)	300 (270–435)	0.738	310 (255–360)	325 (263–465)	0.201
Length of hospital stay (days)	34 (28–40)	38 (28–45)	0.221	36 (29–40)	33 (27–44)	0.597
Postoperative hospital stay (days)	20 (15–26)	21 (17–27)	0.135	20 (15–26)	19 (16–29)	0.529
30 days mortality	1 (2.2)	2 (4.3)	1.000*	0	0	–
Overall complications	9 (20.0)	18(39.1)	0.066	6 (25.0)	7 (29.2)	1.000
Pancreatic fistula^†^	5 (11.1)	8 (17.4)	0.551	3 (12.5)	3 (12.5)	1.000
Postoperative hemorrhage	0	3 (6.5)	0.242*	0	0	–
Biliary fistula	0	2 (4.3)	0.495*	0	0	–
Delayed gastric emptying	3 (6.7)	9 (19.6)	0.119	3 (12.5)	5 (20.8)	0.687
Intra-abdominal infection	1 (2.2)	6 (13.0)	0.111*	1 (4.2)	2 (8.3)	1.000
Cardiopulmonary-related complications	1 (2.2)	1 (2.2)	1.000*	0	0	–
Reoperation	0	1 (2.2)	1.000*	0	0	–
Clavien–Dindo classification			0.180*			0.618
I	5 (11.1)	4 (8.7)		3 (12.5)	1 (4.2)	
II	2 (4.4)	8 (17.4)		2 (8.3)	5 (20.8)	
III	1 (2.2)	3 (6.5)		1 (4.2)	1 (4.2)	
IV	0	1 (2.2)		0	0	
V	1 (2.2)	2 (4.3)		0	0	
Major complications^‡^	2 (4.4)	6 (13.0)	0.267*	1 (4.2)	1 (4.2)	1.000
Postoperative dysfunction^¶^						
Diarrhea	2 (4.5)	3 (6.8)	1.000*	2 (8.3)	1 (4.2)	1.000
Abdominal pain	5 (11.4)	5 (11.4)	1.000	2 (8.3)	4 (16.7)	0.687
Adjuvant therapy			0.559*			0.363
S-1	6 (13.3)	10 (21.7)		3 (12.5)	8 (33.3)	
Gemcitabine	11 (24.4)	6 (13.0)		6 (25.0)	3 (12.5)	
Gemcitabine + capecitabine	3 (6.7)	5 (10.9)		1 (4.2)	2 (8.3)	
Gemcitabine + albumin-bound paclitaxel	5 (11.1)	4 (8.7)		2 (8.3)	2 (8.3)	

Data were expressed as *n* (%) or median (range), unless otherwise marked

^†^According to the International Study Group of Pancreatic Surgery definition (grade B or C)

^‡^Clavien–Dindo classification rating greater than 3 or above

^¶^Patients who died within 1 month were excluded from the analysis

*Fisher exact TEST

Variables with *P* < 0.1 in the univariate analysis were included in the multivariate logistic regression analysis. As shown in Table 4, the surgical approach (SMA-first vs. conventional, OR 0.389, 95% CI 0.152–0.996, *P* = 0.049) was significant in univariate analysis, but only BMI (≥ 24 vs. < 24, OR 3.183, 95% CI 1.004–10.088, *P* = 0.049) was an independent risk factor affecting overall complications in the original cohort in the multivariate analysis.

**Table 4:** Univariate and multivariate logistic regression analysis of overall complications in the original cohort

Variables		Univariate analysis		Multivariate analysis	
		OR (95% CI)	*P* value	OR (95% CI)	*P* value
Age	≥ 60 vs. < 60	0.441 (0.176–1.103)	0.080	0.501 (0.184–1.361)	0.175
Sex	Female vs. male	0.942 (0.378–2.350)	0.898		
Surgical approach	“SMA-first” vs. conventional	0.389 (0.152–0.996)	0.049	0.525 (0.188–1.464)	0.218
BMI	≥ 24 vs. < 24	2.700 (0.948–7.690)	0.063	3.183 (1.004–10.088)	0.049
ASA class	III vs. I/II	0.446 (0.117–1.702)	0.238		
Abdominal surgery	Yes vs. No	1.517 (0.523–4.401)	0.443		
Smoking	Yes vs. No	0.446 (0.117–1.702)	0.238		
Diabetes	Yes vs. No	0.235 (0.028–1.954)	0.180		
Hypertension	Yes vs. No	1.020 (0.345–3.016)	0.971		
Obstructive jaundice	Yes vs. No	1.206 (0.438–3.326)	0.717		
Preoperative biliary drainage	Yes vs. No	0.912 (0.368–2.259)	0.842		
Preoperative CA19-9	> 37 vs. ≤ 37	0.605 (0.217–1.689)	0.338		
Preoperative CEA	> 5 vs. ≤ 5	0.985 (0.310–3.130)	0.979		
Year of surgery	2018–2021 vs. 2014–2017	1.052 (0.428–2.590)	0.912		
TNM staging	II vs. I	2.481 (0.938–6.558)	0.067	2.540 (0.888–7.264)	0.082
	III vs. I	1.323 (0.229–7.638)	0.754	0.992 (0.130–7.592)	0.994
Differentiation	Poor vs. Moderate/Well	1.333 (0.535–3.320)	0.537		
Resection margin	R1 vs. R0	0.875 (0.214–3.586)	0.853		
SMV/PV resection	Yes vs. No	0.773 (0.146–4.098)	0.763		
Conversion	Yes vs. No	7.875 (0.781–79.454)	0.080	7.733 (0.614–97.452)	0.114

*BMI* body mass index, *ASA* American Society of Anesthesiologists, *CA19-9* carbohydrate antigen 19-9, *CEA* carcinoembryonic antigen, *SMV* superior mesenteric vein, *PV* portal vein, *SMA* superior mesenteric artery

### Survival analysis

**Fig. 3:**
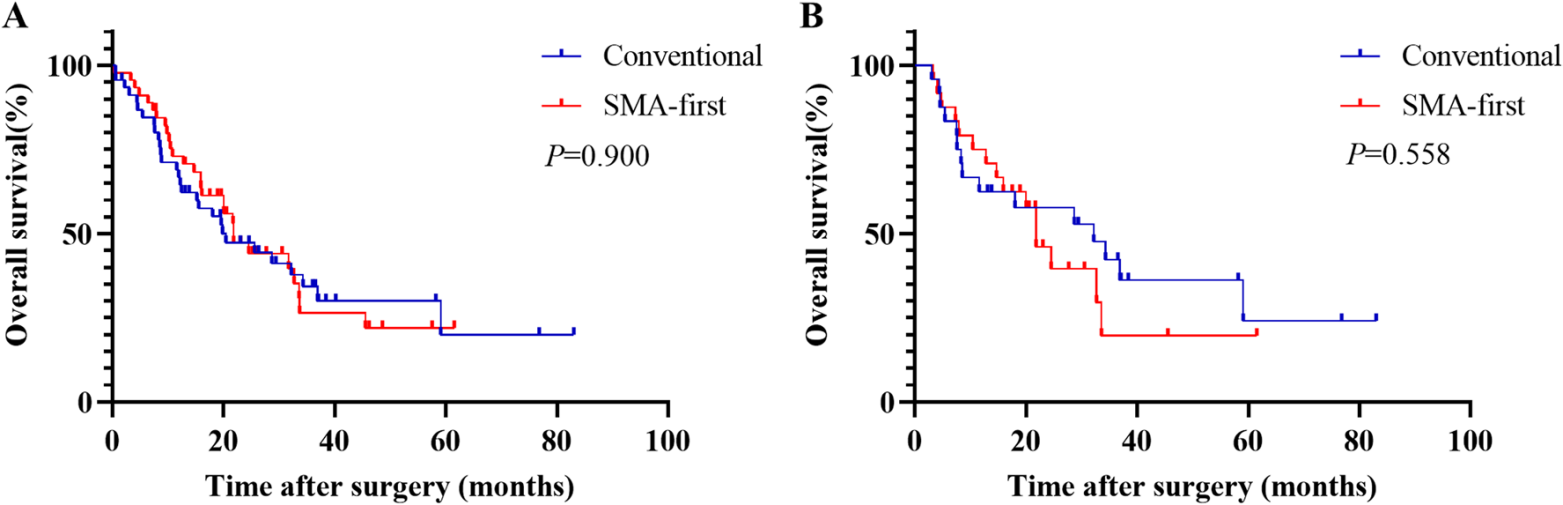
Kaplan–Meier curves comparing overall survival between the SMA-first approach group and the conventional approach group in PDAC patients. **A** Before PSM, and **B** after PSM. *SMA* superior mesenteric artery

**Fig. 4:**
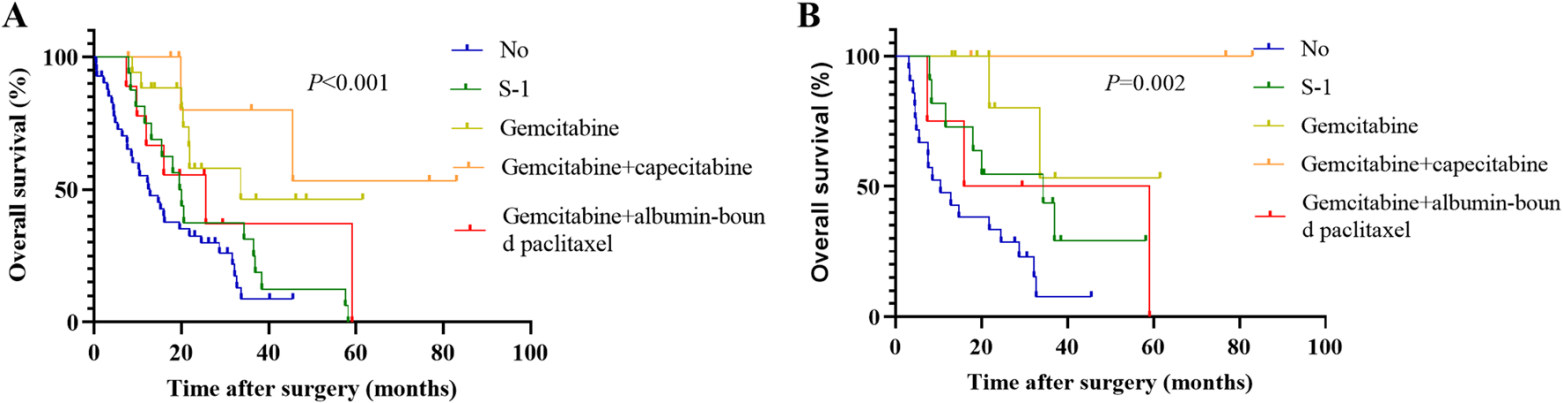
Kaplan–Meier curves comparing overall survival between different adjuvant therapy regimens and non-adjuvant therapy in PDAC patients. **A** Before PSM, and **B** after PSM

During follow-up, 50 patients received adjuvant chemotherapy, of which 16 received S-1 monotherapy, 17 received gemcitabine monotherapy, 8 received gemcitabine plus capecitabine, and 9 received gemcitabine plus albumin-bound paclitaxel (Table 3). The multivariate analysis was conducted by incorporating the variables with *P* < 0.05 from the univariate analysis of COX regression (Table 5). The results indicated that the TNM stage, whether R0 resection was achieved, whether complications occurred, and whether adjuvant therapy was performed were independent risk factors for the OS of PDAC patients in the original cohort (Table 5). The median OS of PDAC patients in the SMA-first approach group was 21.8 months, which was comparable to the conventional approach group with a median OS of 19.8 months (*P* = 0.900, Fig. 3A). In the matched cohort, the median OS was 21.8 months in the SMA-first approach group and 32.2 months in the conventional approach group (*P* = 0.558, Fig. 3B). The OS comparison of the adjuvant therapy groups was statistically different in both the original cohort (*P* < 0.001, Fig. 4A) and the matched cohort (*P* = 0.002, Fig. 4B).

**Table 5:** Univariate and multivariate COX regression analysis of overall survival in the original cohort

Variables		Univariate analysis		Multivariate analysis	
		HR (95% CI)	*P* value	HR (95% CI)	*P* value
Age	≥ 60 vs. < 60	0.987 (0.583–1.669)	0.960		
Sex	Female vs. male	0.672 (0.395–1.142)	0.142		
Surgical approach	“SMA-first” vs. conventional	0.967 (0.574–1.630)	0.900		
BMI	≥ 24 vs. < 24	0.842 (0.445–1.595)	0.598		
ASA class	III vs. I/II	0.934 (0.421–2.073)	0.866		
Abdominal surgery	Yes vs. No	1.772 (0.990–3.172)	0.054		
Smoking	Yes vs. No	1.120 (0.590–2.126)	0.728		
Diabetes	Yes vs. No	0.299 (0.093–0.960)	0.042	0.445 (0.131–1.514)	0.195
Hypertension	Yes vs. No	1.350 (0.757–2.409)	0.309		
Obstructive jaundice	Yes vs. No	1.239 (0.676–2.270)	0.488		
Preoperative biliary drainage	Yes vs. No	1.144 (0.673–1.945)	0.619		
Preoperative CA19-9	> 37 vs. ≤ 37	1.723 (0.870–3.412)	0.119		
Preoperative CEA	> 5 vs. ≤ 5	1.304 (0.655–2.596)	0.450		
Year of surgery	2018–2021 vs. 2014–2017	0.588 (0.337–1.026)	0.061		
TNM staging	II vs. I	2.740 (1.559–4.815)	< 0.001	1.897 (1.015–3.546)	0.045
	III vs. I	1.242 (0.432–3.572)	0.687	2.004 (0.626–6.418)	0.242
Differentiation	Poor vs. Moderate/Well	2.149 (1.273–3.628)	0.004	1.608 (0.873–2.963)	0.128
Resection margin	R1 vs. R0	2.352 (1.098–5.038)	0.028	2.676 (1.148–6.236)	0.023
SMV/PV resection	Yes vs. No	1.692 (0.667–4.291)	0.268		
Conversion	Yes vs. No	0.942 (0.228–3.893)	0.934		
Overall complications	Yes vs. No	1.881 (1.088–3.252)	0.024	1.901 (1.062–3.403)	0.031
Major complications^‡^	Yes vs. No	2.271 (0.969–5.323)	0.059		
Adjuvant therapy	S-1 vs. No	0.417 (0.197–0.882)	0.022	0.396 (0.183–0.860)	0.019
	Gemcitabine vs. No	0.283 (0.124–0.645)	0.003	0.312 (0.135–0.720)	0.006
	Gemcitabine + capecitabine vs. No	0.134 (0.031–0.575)	0.007	0.075 (0.015–0.378)	0.002
	Gemcitabine + albumin-bound paclitaxel vs. No	0.552 (0.227–1.342)	0.190	0.640 (0.256–1.603)	0.341

*BMI* body mass index, *ASA* American Society of Anesthesiologists, *CA19-9* carbohydrate antigen 19-9, *CEA* carcinoembryonic antigen, *SMV* superior mesenteric vein, *PV* portal vein, *SMA* superior mesenteric artery

^‡^Clavien–Dindo classification rating greater than 3 or above

## Discussion

In this study, we described specific surgical steps for a modified SMA-first approach during LPD and its short-term outcomes and long-term survival for treating PDAC patients. Compared with the conventional approach, the SMA-first approach proved to be safe and feasible, although there was only a slight advantage in pathological specimen quality and no significant differences in intraoperative conditions, postoperative complications, and long-term survival.

Nowadays, the application of LPD is increasing year by year, but the therapeutic effect of LPD in pancreatic head cancer is still controversial [5, 20, 21]. In order to improve perioperative safety and prolong patient survival, various pathways have been proposed and continuously improved. The artery-first approach was first proposed by Pessaux [22] and has since been widely used in OPD, where its advantages have been recognized [8]. Laparoscopy has a flexible viewing angle from the caudal to the cephalic side as well as a unique dorsal perspective which facilitates the anatomy and exposure of the SMA, thus the SMA-first approach has also begun to be used in LPD [9–12, 23, 24]. The advantages of the LPD artery-first approach are as follows. First, early exploration of the SMA to determine tumor resectability by artery rather than vein [25]. Second, it can detect and protect the possible aberrant right hepatic artery or common hepatic artery to avoid damage or disconnection that affects the blood supply to the liver [26]. Third, the right nerve and lymphoid tissue with the SMA and CT as the axis can be completely resected to improve the R0 resection rate [12]. Fourth, more in line with the principle of non-contact tumors [27]. Fifth, early dissection of the IPDA during the management of the uncinate process helps reduce intraoperative bleeding and transfusion [28, 29]. Unfortunately, no significant differences were found between the SMA-first approach group and the conventional group in terms of intraoperative bleeding, intraoperative blood transfusion, operative time, and postoperative complication rates and mortality in either the original cohort or matched cohort. The surgical approach was also not an independent risk factor for the eventual occurrence of overall complications. However, the overall complications rate and major complications rate in the SMA-first approach group in the original cohort were indeed less than those in the conventional approach group, and in particular, no postoperative hemorrhage occurred in the SMA-first approach group, which may be the precise identification and accurate treatment of blood vessels during surgery. Some studies suggested that specimen resection time was shorter because the SMA-first approach created a clearer surgical field of view and reduced blood loss [12, 23]. However, only the operative time and gastrointestinal reconstruction time were recorded in our database, and the resection time was not accurately measured, so further records are needed for future comparison.

In 2006, Gockel [30] first proposed the concept of mesopancreas, and in 2012, Adham [31] defined the anatomical structure between the uncinate process and mesenteric vessels as the mesopancreas triangle and proposed the surgical technique of TMpE based on this concept. The anterior triangular boundary is the posterior wall of SMV and PV, the inner boundary is the right edge of the CT and SMA, and the posterior boundary is the surface of the abdominal aorta (AA). The mesopancreas triangle is composed of blood vessels, lymph nodes, nerve fibers, and connective tissue. Inoue et al. [7] also proposed three levels of mesopancreas dissection, and Level 3 clearance should be achieved for invasive pancreatic ductal carcinoma, which means en bloc dissection of mesopancreas and ensuring negative margins for invasive carcinoma. In this study, lymph nodes harvested in the SMA-first approach group were greater than that in the conventional group in both the original and matched cohorts, which may be correlated to TMpE’s removal of vascular tissue including 8p and 9 groups of lymph nodes. The mesopancreas triangle is a site where cancer cells are easily invaded and residual, which is prone to local recurrence of pancreatic head cancer, and TMpE helps prolong patient survival and reduce the rate of local recurrence [32, 33]. The SMA-first approach makes TMpE easier to achieve, which in turn increases the rate of R0 resection [7, 9, 12]. Although no significant difference in R0 resection percentage was found between intergroup in this study, the rate of positive SMA margin was significantly less in the SMA-first approach group than that in the conventional group. In addition, although the SMA-first approach group did not show an advantage in OS, future well-designed trials are needed for further investigation, especially on the local recurrence data.

Nagakawa et al. [13] summarized four common SMA-first approaches for LPD, and Hua et al. [25] further elucidated the advantages and disadvantages of these four approaches. Instead, we used a modified SMA-first approach that combines posterior and anterior approaches. First, we make an enlarged Kocher incision, that is, the posterior approach, to detect whether the SMA is invaded and to be the first to free the SMA origin. Then, after dissecting the pancreatic neck, we completed the mesopancreas resection along the SMA sheath, while dissecting the IPDA in the process, leaving only the specimen attached to the SMV/PV. One of the advantages of this approach is that we can determine the radicality of the tumor at the beginning of the procedure, thus avoiding unnecessary organ dissection. In addition, this approach is suitable for borderline resectable pancreatic head cancer in which the SMV/PV is invaded, where all arterial blood supply has been severed and venous can be safely resected or reconstructed during the final resection. It is worth mentioning that in the SMA-first approach, we introduced, the incidence of postoperative diarrhea is not increased because the left nerve plexus of SMA is preserved.

This study has some shortcomings. First, this study was a single-center retrospective cohort study, and selection bias was unavoidable. Second, this study failed to follow up on the recurrence of patients including local recurrence and distant metastasis, and the effect of the SMA-first approach on long-term survival needs to be further investigated. Third, the modified SMA-first approach described in this study is operationally difficult and is recommended for surgeons who have surmounted the learning curve.

In conclusion, the modified SMA-first approach introduced here can safely and effectively complete laparoscopic uncinate process resection, accurately treat the peri-uncinate process blood vessels and achieve TMpE. Compared with the conventional approach, this approach is safe and feasible. Although there is only a small pathological benefit, such as more lymph nodes harvested and a higher negative percentage of SMA resection margin, with no significant difference in long-term survival, it still has a generalizable value. In the future, more adequate follow-up data, as well as large-sample, prospective trials, are needed to demonstrate the potential benefits of this approach.

### Supplementary Information

Below is the link to the electronic supplementary material.

(DOCX 34 kb)
